# Supplementary material for: Endorsement of COVID-19 misinformation among criminal legal involved individuals in the United States: Prevalence and relationship with information sources
Source: PLoS One. 2024 Jan 5;19(1):e0296752. doi: 10.1371/journal.pone.0296752 (PMC10769018; doi:10.1371/journal.pone.0296752)
Supplement: Attachment — Submitted filename: 25-1-2023 COVID misinformation endorsement PONE-D-22-16444_reviewer.pdf [file pone.0296752.s002.pdf]

PLOS ONE

COVID misinformation endorsement among Americans who are criminal legal involved: Prevalence and relationship with information sources

--Manuscript Draft--

Manuscript Number:	PONE-D-22-16444
Article Type:	Research Article
Full Title:	COVID misinformation endorsement among Americans who are criminal legal involved: Prevalence and relationship with information sources
Short Title:	COVID misinformation in the criminal legal involved population
Corresponding Author:	Xiaoquan Zhao<br>George Mason University College of Humanities and Social Sciences<br>Fairfax, Virginia UNITED STATES
Keywords:	COVID-19; misinformation; criminal legal system involvement; information sources
Abstract:	Criminal legal system involvement (CLI) is a critical social determinant of health that lies at the intersection of multiple sources of health disparities. The COVID-19 pandemic exacerbates many of these disparities, and specific vulnerabilities faced by the CLI population. This study investigated the prevalence of COVID-related misinformation, as well as its relationship with COVID-19 information sources used among Americans experiencing CLI. A nationally representative sample of American adults aged 18+ (N = 1,161), including a subsample of CLI individuals (n = 168), were surveyed in February-March 2021. On a 10-item test, CLI participants endorsed a greater number of misinformation statements (M = 1.88 vs. 1.27) than non-CLI participants, p < .001. CLI participants reported less use of government and scientific sources (p = .017) and less use of personal sources (p = .003) for COVID-19 information than non-CLI participants. Poisson models showed that use of government and scientific sources was negatively associated with misinformation endorsement for non-CLI participants (IRR = .841, p < .001), but not for CLI participants (IRR = .957, p = .619). These findings suggest that building and leveraging trust in important information sources are critical to the containment and mitigation of COVID-related misinformation in the CLI population.
Order of Authors:	Xiaoquan Zhao
	Aayushi Hingle
	Cameron Shaw
	Amy Murphy
	Breonna Riccick
	Rochelle Davidson Mhonde
	Bruce Taylor
	Phoebe Lamuda
	Harold Pollack
	John Schneider
	Faye Taxman
Additional Information:	
Question	Response
**Financial Disclosure**<br><br>Enter a financial disclosure statement that describes the sources of funding for the work included in this submission. Review	FST (#U2CDA050097), JS (1U01MD017414-01), and BT, PAL, HP, and JS (# U2CDA050098) were supported by the U.S. National Institute on Drug Abuse (https://nida.nih.gov/). The funder had no role in study design, data collection and analysis, decision to publish, or preparation of the manuscript.

Powered by Editorial Manager® and ProduXion Manager® from Aries Systems Corporation

the submission guidelines for detailed requirements. View published research articles from *PLOS ONE* for specific examples.<br><br>This statement is required for submission and **will appear in the published article** if the submission is accepted. Please make sure it is accurate.<br><br>**Unfunded studies**<br>Enter: *The author(s) received no specific funding for this work.*<br><br>**Funded studies**<br>Enter a statement with the following details:<br>• Initials of the authors who received each award<br>• Grant numbers awarded to each author<br>• The full name of each funder<br>• URL of each funder website<br>• Did the sponsors or funders play any role in the study design, data collection and analysis, decision to publish, or preparation of the manuscript?<br>• **NO** - Include this sentence at the end of your statement: *The funders had no role in study design, data collection and analysis, decision to publish, or preparation of the manuscript.*<br>• **YES** - Specify the role(s) played.<br><br>* typeset	
**Competing Interests**<br><br>Use the instructions below to enter a competing interest statement for this submission. On behalf of all authors, disclose any competing interests that could be perceived to bias this work—acknowledging all financial support and any other relevant financial or non-financial competing interests.<br><br>This statement is required for submission and **will appear in the published article** if the submission is accepted. Please make sure it is accurate and that any funding sources listed in your Funding Information later in the submission form are also	The authors have declared that no competing interests exist.

declared in your Financial Disclosure statement.<br><br>View published research articles from *PLOS ONE* for specific examples.<br><br>**NO authors have competing interests**<br><br>Enter: *The authors have declared that no competing interests exist.*<br><br>**Authors with competing interests**<br><br>Enter competing interest details beginning with this statement:<br><br>*I have read the journal's policy and the authors of this manuscript have the following competing interests: [insert competing interests here]*<br><br>* typeset	
**Ethics Statement**<br><br>Enter an ethics statement for this submission. This statement is required if the study involved:<br><br>• Human participants<br>• Human specimens or tissue<br>• Vertebrate animals or cephalopods<br>• Vertebrate embryos or tissues<br>• Field research<br><br>Write "N/A" if the submission does not require an ethics statement.<br><br>General guidance is provided below. Consult the submission guidelines for detailed instructions. **Make sure that all information entered here is included in the Methods section of the manuscript.**	The study was approved by NORC's Institutional Review Board. Informed consent was obtained either online or verbally over the phone prior to data collection.

**Format for specific study types**<br><br>**Human Subject Research (involving human participants and/or tissue)**<br>• Give the name of the institutional review board or ethics committee that approved the study<br>• Include the approval number and/or a statement indicating approval of this research<br>• Indicate the form of consent obtained (written/oral) or the reason that consent was not obtained (e.g. the data were analyzed anonymously)<br><br>**Animal Research (involving vertebrate animals, embryos or tissues)**<br>• Provide the name of the Institutional Animal Care and Use Committee (IACUC) or other relevant ethics board that reviewed the study protocol, and indicate whether they approved this research or granted a formal waiver of ethical approval<br>• Include an approval number if one was obtained<br>• If the study involved *non-human primates*, add *additional details* about animal welfare and steps taken to ameliorate suffering<br>• If anesthesia, euthanasia, or any kind of animal sacrifice is part of the study, include briefly which substances and/or methods were applied<br><br>**Field Research**<br><br>Include the following details if this study involves the collection of plant, animal, or other materials from a natural setting:<br>• Field permit number<br>• Name of the institution or relevant body that granted permission	
**Data Availability**<br><br>Authors are required to make all data underlying the findings described fully available, without restriction, and from the time of publication. PLOS allows rare exceptions to address legal and ethical concerns. See the PLOS Data Policy and FAQ for detailed information.	Yes - all data are fully available without restriction

A Data Availability Statement describing where the data can be found is required at submission. Your answers to this question constitute the Data Availability Statement and **will be published in the article**, if accepted.<br><br>**Important:** Stating 'data available on request from the author' is not sufficient. If your data are only available upon request, select 'No' for the first question and explain your exceptional situation in the text box.<br><br>Do the authors confirm that all data underlying the findings described in their manuscript are fully available without restriction?	
**Describe where the data may be found in full sentences. If you are copying our sample text, replace any instances of XXX with the appropriate details.**<br><br>• If the data are **held or will be held in a public repository**, include URLs, accession numbers or DOIs. If this information will only be available after acceptance, indicate this by ticking the box below. For example: *All XXX files are available from the XXX database (accession number(s) XXX, XXX.*).<br>• If the data are all contained **within the manuscript and/or Supporting Information files**, enter the following: *All relevant data are within the manuscript and its Supporting Information files.*<br>• If neither of these applies but you are able to provide **details of access elsewhere**, with or without limitations, please do so. For example:<br><br>*Data cannot be shared publicly because of [XXX]. Data are available from the XXX Institutional Data Access / Ethics Committee (contact via XXX) for researchers who meet the criteria for access to confidential data.*<br><br>*The data underlying the results presented in the study are available from (include the name of the third party*	The data underlying the results presented in the study are available from the NORC JCOIN Methodology and Advanced Analytics Resource Center (MAARC).

and contact information or URL).<br>• This text is appropriate if the data are owned by a third party and authors do not have permission to share the data.<br><br>* typeset	
Additional data availability information:	

Manuscript

Click here to access/download;Manuscript;Misinformation manuscript for Plos One submssion.docx

COVID misinformation in the criminal legal involved population

COVID misinformation endorsement among Americans who are criminal legal involved: Prevalence and relationship with information sources

Xiaoquan Zhao[1], Aayushi Hingle[1], Cameron C. Shaw[2], Amy Murphy[2], Breonna R. Riddick[1], Rochelle R. Davidson Mhonde[1], Bruce J. Taylor[3], Phoebe, A. Lamuda[3], Harold Pollack[4], John Schneider [4,5], & Faye S. Taxman[2]

[1]Department of Communication, George Mason University, Fairfax, Virginia, United States of America
[2]Schar School of Public Policy, George Mason University, Fairfax, Virginia, United States of America
[3]NORC at the University of Chicago, Chicago, Illinois, United States of America
[4]Crown Family School of Social Work, Policy, and Practice, University of Chicago, Chicago, Illinois, United States of America
[5]Department of Medicine, University of Chicago, Chicago, Illinois, United States of America

Corresponding author: Xiaoquan Zhao, email: xzhao3@gmu.edu

Author contributions: Conceptualization: XZ and FST; Data curation: BJT, PAL, HP, and JS; Formal analysis: XZ and FST; Funding acquisition: BJT, HP, JS, and FST; Investigation: AH, CCS, AM, BRR, and RRDM; Writing – original draft: XZ, AH, CCS, AM, FST; Writing – review & editing: BRR, RRDM, BJT, PAL, HP, & JS

Abstract

Criminal legal system involvement (CLI) is a critical social determinant of health that lies at the intersection of multiple sources of health disparities. The COVID-19 pandemic exacerbates many of these disparities, and specific vulnerabilities faced by the CLI population. This study investigated the prevalence of COVID-related misinformation, as well as its relationship with COVID-19 information sources used among Americans experiencing CLI. A nationally representative sample of American adults aged 18+ ($N = 1,161$), including a subsample of CLI individuals ($n = 168$), were surveyed in February-March 2021. On a 10-item test, CLI participants endorsed a greater number of misinformation statements ($M = 1.88$ vs. 1.27) than non-CLI participants, $p < .001$. CLI participants reported less use of government and scientific sources ($p = .017$) and less use of personal sources ($p = .003$) for COVID-19 information than non-CLI participants. Poisson models showed that use of government and scientific sources was negatively associated with misinformation endorsement for non-CLI participants (IRR = .841, $p < .001$), but not for CLI participants (IRR = .957, $p = .619$). These findings suggest that building and leveraging trust in important information sources are critical to the containment and mitigation of COVID-related misinformation in the CLI population.

Introduction

Misinformation poses a significant threat to effective public health responses to the COVID-19 pandemic [1–3]. Ample research has focused on the prevalence, impact, and correction of COVID-19 misinformation in the general population [4,5]. Less attention has been paid to misinformation among minoritized and marginalized communities [1]. To the best of our knowledge, no research has specifically examined the presence and dissemination of misinformation among individuals with criminal legal system involvement (CLI), a population that has traditionally faced significant health disparities. In this study, we assess the prevalence of COVID-19 misinformation among a nationally representative sample of CLI and non-CLI individuals in the United States (U.S.). The associations between sources used for COVID-19 information and misinformation endorsement within CLI vs. non-CLI groups are also investigated.

CLI as a Social Determinant of Health

CLI refers to current or prior experience of involvement in the criminal legal system such as arrest, incarceration, probation or parole, or another legal status. As a unique social determinant of health, CLI is intertwined with other structural influences on health, such as racial discrimination, poverty, food and housing insecurity, education quality, health care access, and neighborhood environments [6]. CLI impacts health both directly via the conditions of legal involvement and indirectly through CLI's impact on other social determinants of health such as economic well-being. Adults on probation, for example, often face difficulties in securing employment, food, and housing, which place them at high risk for both recidivism and adverse health outcomes [7].

Individuals with a history of incarceration experience worse health outcomes compared to the general population. Incarcerated people are more likely to have high blood pressure, asthma, cancer, arthritis, and infectious diseases, such as tuberculosis, hepatitis C, and HIV [8]. Half of all people in state and federal prisons have a chronic medical condition [9]. The National Institute on Drug Abuse or (NIDA) estimates that 65% of individuals in prison and over 70% of people with criminal legal involvement have a substance use disorder (SUD) [10]. Winkelman and colleagues found that more than half of individuals with opioid use disorder have had contact with the criminal legal system and that as the level of opioid use increased, involvement in the justice system also increased [11]. More than one-third (37% to 44%) of incarcerated individuals report ever being diagnosed with a mental health disorder, with 14% of individuals in prisons and 26% in jails having experienced serious psychological distress [12]. In the criminal legal system, 24% to 34% of women and 12% to 15% of men have co-occurring SUD and mental health disorders [13–15]. Using data from the 2009 National Survey on Drug Use and Health (NSDUH), Vaughn and colleagues (2012) found that individuals who self-disclosed being on probation or parole were two to five times more likely to report use of an illicit substance, such as heroin or cocaine, in the past year. They were also more likely to report having an SUD and having received treatment for substance use or mental health disorder in the past year [16].

CLI Disparities during the Pandemic

Health disparities faced by the CLI population place individuals at heightened risk for both infection and death during the COVID-19 pandemic. Correctional environments present a number of factors that increase risk of COVID-19 exposure to both residents and staff [17], including an aging incarcerated population with high rates of underlying and chronic health conditions; overpopulated, confined spaces and unsanitary conditions; limited healthcare

capacity; as well as continuous movement within, into, and out of the facilities by residents, staff, attorneys, and other visitors [17–20]. The UCLA COVID-19 project shows that, as of the current writing, at least 599,617 COVID-19 cases among incarcerated individuals have been reported, including 3,099 deaths, plus an additional 207,438 cases and 308 deaths among carceral institution staff [21]. Data from early in the pandemic indicated that incarcerated individuals were 5.5 times more likely to be infected with COVID-19 and 3 times more likely to die from it than the rest of the U.S. population (Saloner et al., 2020).

The disproportionate effect of incarceration on marginalized communities defined by race, ethnicity, and socioeconomic status is well established [18,22,23]. For Black, Indigenous, and other people of color (BIPOC), systemic barriers to equitable healthcare are a continuous concern [24–26]. These disparities are reflected in higher rates of COVID-19 hospitalizations and mortality among BIPOC communities [27–29]. In general, BIPOC communities are 2.5 times more likely to be hospitalized and 1.7 times more likely to die from COVID-19 than White individuals. Hospitalization and death rates are slightly higher for Black individuals than Hispanic or Latinx individuals, and Native or Indigenous communities face the highest rates of infection and death [30]. The complex nexus of racial, socioeconomic, and CLI disparities poses a unique challenge to health equity during the pandemic.

In analyzing COVID-19 protective behaviors among various populations, Schneider et al. [31] indicate that CLI individuals are overall less likely to report protective behaviors, which may be a result of employment factors, limited access to personal protective equipment, and crowded housing or homelessness. Notably, CLI individuals are less likely to use face coverings but more likely to be tested than other populations of the study. The authors note that testing behaviors may be a direct effect of involuntary confinement in crowded carceral settings or

needed for access to community services. This suggests that the CLI population's responses to the COVID-19 pandemic are profoundly shaped by the social, environmental, and legal factors in their lived experiences.

COVID-19 Misinformation

Misinformation about COVID-19 is so widespread and influential that the World Health Organization (WHO) declared that the pandemic is concurrently an "infodemic" (WHO, 2020). Misinformation is defined as "information that is false, inaccurate, or misleading according to the best available evidence at the time" [1]. Some consider misinformation a meta-risk during the COVID-19 pandemic as it interacts with and complicates perceptions about the original risk [33]. The threat of a deadly virus, coupled with evolving uncertainty and increasing politicization of the pandemic, has fueled rampant growth and dissemination of misinformation. False information tends to diffuse "farther, faster, deeper, and more broadly" than accurate information [34]. An early assessment during the pandemic reported that online messages from medical and public health authorities, such as the WHO and Centers for Disease Control (CDC), generated much less public engagement than platforms hosting misinformation and conspiracy theories [35]. There is evidence that this pattern has persisted as the pandemic continues [36].

Prevalent misinformation can influence public perceptions and pandemic response, with strong documented associations between COVID-19 misinformation exposure and maladaptive knowledge, beliefs, and behaviors [3,37–39]. A study in Hong Kong found that exposure to misinformation about smoking and drinking being protective factors against COVID-19 was associated with increased use of both substances among current users [40]. In the U.S., endorsing misinformation about COVID-19 was found to undermine prevention self-efficacy, which could in turn negatively impact preventive behaviors [41]. A study from Canada found that exposure to

COVID-19 misinformation was associated with clinically significant anxiety disorder among men [42]. Experimental data from Ireland showed that a single exposure to fabricated news stories about COVID-19 could generate measurable effects on protection-relevant behaviors [37]. Given the critical importance of vaccination to combat against COVID-19, misinformation about the vaccines is particularly detrimental [43]. A recent randomized controlled trial found that exposure to misinformation could decrease vaccination intent by 6.2% in the U.K. and 6.4% in the U.S. among those who had previously indicated intention to get vaccinated [2].

Recent data from the COVID States Project indicate that belief in vaccine misinformation is intricately related to sociodemographic variables in the U.S. [3]. Men, 35 to 44 -year-olds, African Americans, Hispanic or Latinx/e people, parents of young children, those with lower education, and Republicans are more likely to hold vaccine misperceptions. Moreover, the relationships between misinformation acceptance and socioeconomics appear to have evolved over time. For example, those with high education and income were among the most likely to accept vaccine myths early in the pandemic. The same groups, however, are now among the least likely to endorse vaccine misinformation [3].

Although some sociodemographic differences in misinformation have varied across studies [cf. 41], the partisan divide on COVID-19 misinformation acceptance has been demonstrated repeatedly [41,44,45]. In general, individuals leaning Republican are more likely to hold COVID-19 misperceptions than those leaning Democratic. Furthermore, the ability to withstand exposure to misinformation appears to vary across gender, income, and racial and ethnic groups [2]. To the best of our knowledge, no research has examined the prevalence of COVID-19 misinformation in the CLI population, although studies of general COVID-19

knowledge and beliefs in this population point to the possibility of increased vulnerability [46,47].

COVID-19 Information Sources

The COVID-19 pandemic happened at a time of unprecedented technological advancement and fragmentation in the informational environment [48]. A long research tradition in health communication focuses on the relationship between the usage of different information sources and health attitudes and behaviors [49–51]. The plethora of information sources available today suggests that differential reliance and use of sources may lead to distinctive patterns of information gain, including the reception and acceptance of misinformation, by different populations. It is difficult to accurately estimate the amount of misinformation circulated in different sources. One analysis of fact-checked misinformation in 2020 showed that the vast majority of the identified misinformation appeared on social media (88%), followed by TV (9%), published news outlets (8%), and other websites (7%) [52]. However, these findings have to be interpreted with caution because the sampled misinformation represented a particularly influential category (i.e., false ideas widespread enough to catch the attention of fact-checking organizations). Moreover, high prevalence of misinformation on social media does not mean that the net effect of social media use is necessarily detrimental because social media also include critical outlets for accurate health information. On this latter point, there is research showing that participants in the U.S. receiving more COVID-19 information online report more frequent efforts to engage in all types of preventive behaviors [53], although counter evidence also exists [45].

A few studies have examined the relationship between media source usage and COVID-19 misinformation endorsement. A national probability survey conducted in the U.S. in early

2020 showed low perceived lethality of the coronavirus and high endorsement of misinformation [45]. Moreover, exposure to mainstream broadcast and print media was correlated with accurate risk perceptions and less belief in misinformation, whereas exposure to conservative media was correlated with higher levels of misinformation. Another nationally representative survey conducted a few months later replicated the findings on the different associations between partisan media use and misinformation endorsement [41]. It showed that people relying on conservative media sources tended to score higher on COVID-19 misinformation. In contrast, those using liberal sources, mainstream print, or social media as primary sources of COVID-19 news tended to score lower. Additional studies have produced mixed findings regarding specific information sources. A multinational study, for example, found that exposure to traditional media (e.g., television, radio, newspapers) was associated with lower belief in COVID-19 conspiracy theories and misinformation [54]. Conversely, another study done in the U.S. found that higher news consumption through traditional media was associated with lower knowledge and more fake news beliefs [55].

It is important to note that trust is a critical determinant of the types of information CLI individuals seek out for health and how information consumption may alleviate or deepen disparities depending on the information obtained [56,57]. Trust is often determined by the intersections of personal and social identities such as race, ethnicity, socioeconomic status, and region, to name a few [58–60]. Individuals involved in the criminal legal system often turn to supplemental or nontraditional sources, such as peer navigators, for health information because of general distrust in and negative experiences with the government and medical establishment [61,62]. A recent study of CLI women found that Black women chose TV as their most trusted source of information regarding COVID-19, while White women chose government or social

service agencies as their most trusted sources [63]. Additionally, 15% of the women studied reported not trusting any sources of information. Trust, or the lack thereof, factors significantly in information consumption among the CLI population during the pandemic. For racially minoritized groups in this population, the pattern and level of trust in information sources most likely reflect the dual influence of structural racism and historical mistreatment [64].

Current Study

Although COVID-19 misinformation has garnered significant attention during the pandemic, research has only begun to examine this phenomenon from a health equity perspective. This study focuses on COVID-19 misinformation among a general population inclusive of a unique high-risk subpopulation – individuals with criminal legal system involvement. Our first aim is to assess the prevalence of misinformation among CLI individuals as compared to non-CLI individuals. Our second aim is to assess the relationship between misinformation endorsement and patterns of information source usage within each of these two groups. The overarching goal is to shed light on misinformation as a unique form of disparity affecting CLI communities during the pandemic and the extent to which use of information sources may be associated with the genesis and persistence of this disparity.

Method

Survey

Data used in the study came from the AmeriSpeak Omnibus survey conducted by NORC at the University of Chicago. AmeriSpeak is a probability-based panel of about 35,000 households recruited using area probability and address-based sampling. The panel provides sample coverage of approximately 97% of the U.S. household population. The AmeriSpeak Omnibus survey is conducted monthly with a nationally representative sample of adults aged 18

and older drawn from the AmeriSpeak panel with probability sampling based on sex, age, race/ethnicity, and education. Most AmeriSpeak households participate in surveys online through either conventional internet connection or smartphone access, and non-internet households can participate in AmeriSpeak surveys by telephone. The questionnaire used for this study was fielded in February and March of 2021 (N = 1,161). Survey invitations were sent out by email to the selected panel members. Those who did not respond to the initial invitation were contacted multiple times by email and phone. The survey was offered in both English and Spanish. Respondents received an incentive worth $4 for their participation. The overall response rate for this survey was 11.1% (37% panel recruitment response rate multiplied by 30% within-panel study-specific response rate). The study protocol was approved by NORC's Institutional Review Board. Informed consent was obtained either online or verbally over the phone prior to data collection.

Measures

The survey covered a wide range of questions, including a module specifically designed for this study. In this module, respondents were presented with 10 statements about COVID-19 (six false and four true) and asked to indicate each as true or false (see Table 3 for items). The statements were all based on misinformation extracted from earlier focus group research with a sample of CLI individuals recruited from several central states in the U.S. Methodological details and findings from the focus group research are presented elsewhere[65]. To minimize straight-lining in responses, several of the misinformation items were reworded to be factual/accurate in nature (e.g., "I cannot get COVID-19 by getting tested for it"). Responses to factual statements were reverse coded to be consistent with the coding of misinformation statements. The total

number of incorrect responses across the 10 items was tallied as a measure of overall misinformation endorsement.

Respondents reported the sources from which they obtained most of their information about COVID-19 in the past month (yes or no). Fifteen sources were shown and we grouped the sources based on joint consideration of (a) the nature of each source, (b) commonly used categorization schemes in previous research [e.g., 41,66], and (c) an exploratory factor analysis using Mplus (v.8) and weighted least squares mean and variance estimator (WLSMV) given the categorical data. In the end, five source categories were created: (a) government and science: federal government, local and state government, and scientific journals; (b) mainstream and news media: broadcast TV, cable TV, national print, local print, radio news, and online news; (c) social media; (d) personal sources: personal networks and employer; and (e) community sources: community organizations, church, and other. An index for each source category was created by adding positive responses (yes) within the category, and the indexes correlated mildly with one another (max. $r = .414$).

To measure criminal legal system involvement (CLI), the survey asked whether the respondent had ever been “convicted of any misdemeanor or felony crime” or “been incarcerated in jail or prison.” A positive response to either indicated CLI.

Demographics and other background variables included biological sex, age, race/ethnicity, education, income, marital status, employment status, and political party identification. Most of these variables were gathered by the AmeriSpeak panel and updated annually. Employment status and political party identification were asked in the current survey to ensure the information was most up to date.

Analysis

Descriptive analysis was performed on all study variables both for the full sample and for subsamples stratified by CLI. We used chi-square tests to compare CLI and non-CLI participants on sample characteristics and information source usage. For misinformation endorsement, we employed logistic regression for individual items and Poisson regression for the total number of misinformation statements endorsed. We ran two sets of Poisson models, one for the full sample, the other for CLI and non-CLI subsamples. The full sample models included CLI status, source usage, and their interactions (one interaction at a time to minimize the threat of multicollinearity), in addition to demographic controls. The interaction terms were constructed using mean-centered source usage variables. The subsample models (stratified by CLI status) only included source usage variables and demographic controls. Unless otherwise noted, all analyses were weighted to align with national benchmarks and account for selection probabilities and non-response in sampling. Missing data were minimal (max = 1.1% in any analysis) and handled by listwise deletion. Multicollinearity diagnostics did not reveal any cause for concern in any of the regression models. The software package used for analyses was IBM SPSS v.28.

Results

Sample characteristics

Sample characteristics are summarized in Table 1. Compared to non-CLI participants, CLI participants were more likely to be male, 30 to 59 years in age, and non-Hispanic Black; more likely to report less than college education and the lowest level of income; less likely to be currently married; less likely to be Democrats and more likely to be Independent or neutral in political leaning.

Table 1. Sample characteristics

	**Total Unweighted %**	**Non-CLI Weighted %**	**CLI Weighted %**	**p**

	**N = 1,161**	**N = 989**	**N = 168**	
Sex				.009
Male	47.8	46.7	57.5	
Female	52.2	53.3	42.3	
Age				.016
18-29	16.7	21.6	15.7	
30-34	30.4	23.8	31.3	
45-59	23.5	23.6	29.1	
60+	29.4	31	23.9	
Race/Ethnicity				.045
NH White	63.4	64.0	54.7	
NH Black	13.2	11.2	16.6	
Hispanic	16.9	16.7	16.9	
Other	6.5	8.1	11.8	
Education				<.001
Less than HS	4.8	9.5	11.8	
HS graduate	16.8	25.7	39.9	
Some college	43.9	27.9	26.6	
Bachelor’s degree	19.6	20.9	13.8	
Graduate degree	14.8	16.1	8.0	
Income				<.001
< $30k	22.9	22.2	36.2	
$30K - <$60K	28.6	29.5	23.2	
$60K - <$100K	26.6	23.8	27.8	
$100k+	21.9	24.6	12.8	
Marital status				<.001
Currently married	48.2	50.1	37.0	
Never married	24.9	25.6	26.1	
Divorced	11.4	10.0	18.9	
Other	15.5	14.3	18.0	
Employment status				.206
Currently employed	63.9	58.0	63.4	
Other	36.1	42.0	36.6	
Political party/leaning				.018
Democrat	37.8	35.3	28.0	
Lean Democrat	10.6	11.2	9.1	
Independent/None	15	14.2	23.5	
Lean Republican	10.1	10.5	12.4	
Republican	26.0	28.4	26.9	

Note. CLI = criminal legal involved.

Misinformation endorsement

Endorsement levels for the 10 misinformation statements among CLI and non-CLI participants are presented in Table 2. About three quarters of the CLI participants (72.6%) and 66.5% of non-CLI participants endorsed at least one misinformation statement, a difference that did not reach statistical significance, $\chi^2 = 2.448$, df = 1, p = .130. The average number of misinformation statements endorsed by CLI participants was 1.88, compared to 1.27 by non-CLI participants. This difference was statistically significant, t = 5.123, df = 1,155, p < .001, d = .427.

Table 2. Endorsement of misinformation

**Statement**	**% Endorsing Non-CLI**	**% Endorsing CLI**	**Un-adj. OR (95%CI)**	**Adj. OR (95%CI)**
People with certain blood types will never get COVID-19.	4.6	9.6	**2.218 (1.224, 4.019)**	**2.069 (1.109, 3.861)**
African Americans are less likely to get COVID-19 compared to other racial groups.	6.8	5.5	.798 (.394, 1.617)	.746 (.360, 1.547)
A vaccine for COVID-19 is already available. (R)	6.1	14.1	**2.497 (1.505, 4.143)**	**2.363 (1.385, 4.032)**
I cannot get COVID-19 by getting tested for it. (R)	35.4	44.1	**1.406 (1.024, 1.987)**	1.075 (.752, 1.537)
If I eat right, exercise, and take good care of my body, I don’t need to worry about getting COVID-19.	7.9	12.8	**1.702 (1.025, 2.826)**	1.488 (.871, 2.543)
COVID-19 is a scheme for rich people and big companies to make money off of the testing and vaccines.	13.4	21.9	**1.804 (1.198, 2.717)**	**1.690 (1.084, 2.636)**
The COVID-19 vaccines are coming out so fast because they have not been carefully tested.	33.7	52.2	**2.917 (1.575, 3.064)**	**2.131 (1.486, 3.054)**
Taking HIV/AIDS medications would protect me from COVID-19.	2.2	5.3	**2.440 (1.099, 5.415)**	1.844 (.798, 4.445)
A person with COVID-19 who wears a mask can still spread COVID-19 to other people. (R)	11.5	12.1	1.065 (.644, 1.759)	.812 (.479, 1.376)
If someone I live with has COVID-19, it increases my chance of getting COVID-19. (R)	5.4	10.2	**1.984 (1.121, 3.512)**	1.659 (.911, 3.018)
	**M (SD)**	**M (SD)**	**Un-adj. IRR**	**Adj. IRR (95%CI)**
Total # of statement endorsed	1.27 (1.34)	1.88 (1.87)	**1.475 (1.298, 1.676)**	**1.299 (1.139, 1.481)**

Note. CLI = criminal legal involved. OR = odds ratio. IRR = incidence rate ratio. CI = confidence interval. Adj. = adjusted. R = reverse coded. Adjusted OR controlled for gender, age, race/ethnicity, education, income, marital status, employment status, and political party/leaning.

For most statements, the endorsement rate was relatively low in both subsamples (around or below 15%). Two statements, however, received strong endorsement from both CLI and non-CLI participants: “I cannot get COVID-19 by getting tested for it” (reverse coded; 44.1% for CLI and 35.4% for non-CLI) and “The COVID-19 vaccines are coming out so fast because they have not been carefully tested” (52.2% for CLI and 33.7% for non-CLI). The endorsement level was significantly higher among CLI participants than among non-CLI participants for 8 statements in bivariate logistic regression (see un-adj. ORs in Table 2). In multivariable logistic regression controlling for an array of demographic, socioeconomic, and political background variables, four of the differences remained significant (see adj. ORs in Table 2). These included: “People with certain blood types will never get COVID-19;” “A vaccine for COVID-19 is already available (reverse coded);” “COVID-19 is a scheme for rich people and big companies to make money off of the testing and vaccines;” and “The COVID-19 vaccines are coming out so fast because they have not been carefully tested.” In terms of total number of statements endorsed, Poisson regression revealed significant difference by CLI status in both bivariate and multivariable analyses (see bottom of Table 2).

COVID-19 information sources

Table 3 presents descriptive data on COVID-19 information sources by CLI status. For CLI participants, the top five information sources were broadcast TV (56.8%), cable TV (47.9%), personal networks (31.0%), online news (30.8%), and state or local government (28.4%). For non-CLI participants, the top five were broadcast TV (47.2%), personal networks (40%), cable TV (39.6%), State or local government (38.9%), and federal government (33.7%). For broadcast TV ($p = .024$), cable TV ($p = .042$), personal networks ($p = .026$), and state and local government ($p = .010$), the use rates between CLI and non-CLI groups were significantly

different. CLI participants reported greater use of broadcast and cable TV but less use of state or local government and personal networks than non-CLI participants.

Table 3. COVID information sources used last month

	**Weighted % non-CLI**	**Weighted % CLI**	**p**
Government and science (>=1)	**49.8**	**39.9**	**.017**
State or local government	**38.9**	**28.4**	**.010**
Federal government	33.7	27.2	.110
Scientific journal	11.4	8.3	.286
Mainstream/news media (>=1)	77.6	81.5	.256
Local print	18.2	16.1	.586
National print	12.8	10.7	.528
Broadcast TV	**47.2**	**56.8**	**.024**
Cable TV	**39.6**	**47.9**	**.042**
Radio news	18.1	16.1	.586
Online news	31.5	30.8	.929
Social media	27.1	25.6	.708
Social media	27.1	25.6	.708
Personal sources (>=1)	**47.5**	**35.1**	**.003**
Employer	16.5	11.8	.138
Personal networks	**40.0**	**31.0**	**.026**
Community sources (>=1)	11.5	9.5	.447
Church	5.1	3.6	.559
Community Organization	3.9	4.7	.672
Other	4.6	5.9	.434

Note. CLI = criminal legal involved.

After grouping these sources into categories, two categories showed significant differences by CLI status. CLI participants reported less use of government and scientific sources (p = .017) and less use of personal sources (p = .003) than non-CLI participants. The two groups did not differ significantly on their use of mainstream and news media (p = .256), social media (p = .708), and community sources (p = .447).

Misinformation endorsement and information sources

Table 4 presents the results of the Poisson regression models predicting total number of misinformation statements endorsed in the full sample using CLI, source usage, and their

interactions (one at a time) as key predictors while controlling for sociodemographic and political background variables. CLI emerged a positive predictor of misinformation endorsement for participants not using the sources involved in the interaction in each of the five models (all IRR > 1.210, all p < .006). Additionally, two of the interactions were significant, CLI with mainstream/news media use (IRR = .785, p < .001) and CLI with social media use (IRR = .713, p = .031). A third interaction between CLI and personal sources bordered on significance (IRR = .802, p = .051). In all three cases, greater usage of the sources was associated with less discrepancy in misinformation endorsement between CLI and non-CLI participants. From a different angle, the interaction also showed that the association between source usage and misinformation endorsement was weaker or more negative among CLI participants than among non-CLI participants.

Table 4. Interactions between CLI status and sources of COVID information in predicting total number of misinformation statements endorsed (full sample analysis)

	**IRR**	**95% CI**		**Sig.**
		**Lower Bound**	**Upper Bound**	
CLI	**1.233**	**1.066**	**1.425**	**0.005**
Government and science	**0.877**	**0.813**	**0.946**	**<.001**
Interaction	0.92	0.779	1.086	0.324
CLI	**1.21**	**1.056**	**1.386**	**0.006**
Mainstream/News	**1.059**	**1.009**	**1.112**	**0.02**
Interaction	**0.785**	**0.707**	**0.871**	**<.001**
CLI	**1.266**	**1.108**	**1.447**	**<.001**
Social media	1.063	0.929	1.216	0.374
Interaction	**0.713**	**0.524**	**0.97**	**0.031**
CLI	**1.22**	**1.06**	**1.404**	**0.006**
Personal	0.935	0.847	1.032	0.18
Interaction	0.802	0.643	1.001	0.051
CLI	**1.282**	**1.123**	**1.464**	**<.001**
Community	**1.278**	**1.112**	**1.468**	**<.001**
Interaction	1.038	0.799	1.348	0.779

Note. CLI = criminal legal involved. IRR = incident rate ratio. CI = confidence interval. Each model included one CLI by source interaction, with other source usage variables controlled for. Additional control variables included gender, age, race/ethnicity, education, income, marital status, employment status, and political party/leaning . Control variables in the models are not reported.

Table 5 presents separate Poisson models for CLI and non-CLI subsamples to further examine the relationship between source usage and misinformation endorsement within each group. In the CLI model, use of mainstream/news media (IRR = .844, p = .002) was negatively associated with, and use of community sources (IRR = 1.569, p = .002) was positively associated with misinformation endorsement. In the non-CLI model, use of government and scientific sources (IRR = .841, $p < .001$) were negatively associated with, and use of community sources (IRR = 1.249, p = .002) were positively associated with misinformation endorsement. Use of social media or personal sources were unrelated to misinformation endorsement for either group (all $p > .105$). Use of government and science sources was not associated with misinformation endorsement for the CLI group (p = .619) and use of mainstream/news media was not associated with misinformation endorsement for the non-CLI group (p = .085).

Table 5

Associations between total number of misinformation statements endorsed and sources of COVID information for CLI and non-CLI subsamples

	Non-CLI				CLI			
		95% CI				95% CI		
	IRR	Lower Bound	Upper Bound	Sig.	IRR	Lower Bound	Upper Bound	Sig.
Male	0.894	0.795	1.006	0.064	0.891	0.688	1.155	0.384
Oder age	**0.89**	**0.837**	**0.947**	**<.001**	**0.712**	**0.621**	**0.816**	**<.001**
Education	**0.847**	**0.8**	**0.898**	**<.001**	0.87	0.756	1.001	0.051
Income	0.991	0.93	1.056	0.787	0.98	0.835	1.149	0.8
NH-Black	**2.277**	**1.885**	**2.751**	**<.001**	**1.935**	**1.261**	**2.971**	**0.003**
Hispanic	**1.609**	**1.382**	**1.872**	**<.001**	0.966	0.682	1.369	0.846
Other race/ethnicity	1.211	0.956	1.533	0.112	1.213	0.769	1.913	0.406
Leaning Republican	**1.142**	**1.097**	**1.19**	**<.001**	**1.341**	**1.212**	**1.484**	**<.001**
Married	0.926	0.81	1.059	0.261	1.22	0.916	1.627	0.174
Employed	1.035	0.909	1.179	0.601	1.021	0.749	1.392	0.895
Government and science	**0.841**	**0.777**	**0.909**	**<.001**	0.957	0.805	1.138	0.619
Mainstream/news	1.045	0.994	1.099	0.085	**0.844**	**0.757**	**0.942**	**0.002**
Social media	1.045	0.912	1.198	0.528	0.772	0.535	1.114	0.167
Personal	0.931	0.841	1.03	0.163	0.813	0.632	1.045	0.105
Community	**1.249**	**1.085**	**1.438**	**0.002**	**1.569**	**1.184**	**2.08**	**0.002**
	LR Chi-Square = 298.35, df = 15, p < .001				LR Chi-Square = 106.91, df = 15, p < .001			

Note. CLI = criminal legal involved. IRR = incident rate ratio. CI = confidence interval. NH = non-Hispanic. Adj. = adjusted. LR = likelihood ratio.

Several covariates also emerged significant in the analyses. In the CLI model, misinformation endorsement was negatively associated with younger age (IRR = .712, $p < .001$) and positively associated with being non-Hispanic Black (IRR = 1.935, $p = .003$) and leaning Republican in political orientation (IRR = 1.341, $p < .001$). In the non-CLI model, negative covariates of misinformation endorsement included younger age (IRR = .890, $p < .001$) and higher education (IRR = .847, $p < .001$), and positive covariates included being non-Hispanic Black (IRR = 2.277, $p < .001$), being Hispanic (IRR = 1.609, $p < .001$), and leaning Republican in political orientation (IRR = 1.142, $p < .001$).

Discussion

This study examined the prevalence of misinformation and its relationship with COVID-19 information sources among CLI vs. non-CLI individuals in the U.S. Our nationally representative survey data showed that the rate of misinformation endorsement was indeed higher among CLI respondents than among their non-CLI counterparts. CLI respondents reported greater acceptance on nine of the ten misinformation items assessed in this study. Among the nine items, eight showed significant differences between the CLI and non-CLI groups in unadjusted analysis, and four remained significant in analyses adjusting for an array of sociodemographic and political background variables. When the total number of endorsed misinformation items was treated as the outcome, CLI status consistently emerged as a significant positive predictor in a series of models with varying sets of control variables (see Tables 3 and 4). It appears clear that the CLI population is indeed more prone to endorsing COVID-19 misinformation than is the non-CLI population in the U.S.

An important caveat for these results is that the misinformation items used in this study were based on earlier qualitative research with CLI participants [65]. It is thus possible that this

specific set of items might have an inherent bias toward greater endorsement among CLI respondents. While this is a reasonable possibility, it is notable that most, if not all, of the misinformation tested in this study is also widely circulated in the general population. For example, the two statements with the highest rates of endorsement in our data were about rushed vaccine development and contracting COVID-19 through testing. These misperceptions are widely documented in previous general population studies and continuing misinformation surveillance efforts [3,41,45]. We saw substantial gaps between the CLI and non-CLI groups on these two items, with the one about vaccine development reaching almost 20%. This suggests that the greater prevalence of misinformation among the CLI population is a real and broad phenomenon that is not likely to be entirely driven by the specific set of items used in this study.

Previous research, both before and during the pandemic, suggests that health knowledge and beliefs, misperceptions included, are intricately associated with the sources people use to obtain health information [3,49,50]. Our data showed different patterns of information source usage among CLI and non-CLI individuals. CLI individuals were significantly less reliant on government/scientific and personal sources, and more reliant on broadcast and cable TV, for COVID-19 information compared to their non-CLI peers. Moreover, for most of the information sources, fewer members of the CLI group reported usage than the non-CLI group, although most of the differences did not reach statistical significance.

A few patterns are notable in the relationship between source usage and misinformation endorsement for CLI and non-CLI respondents. First, use of government and scientific sources was associated with reduced misinformation endorsement among non-CLI individuals, but not among CLI individuals. This difference did not emerge as significant in the interaction analysis, but was fairly visible in the subsample analyses. The IRR of government/scientific sources for

the non-CLI subsample was well below 1 (.841 to be exact), indicating a negative association, while the IRR for the CLI subsample was virtually indistinguishable from 1 (.957 to be exact), indicating no relationship. Second, mainstream and news media use was negatively associated with misinformation endorsement for the CLI subsample. For the non-CLI subsample, the association was in the opposite direction, although not significant. The difference between the CLI and non-CLI subsamples was nevertheless significant by virtue of the interaction analysis. Third, the use of social media was not significantly associated with misinformation endorsement in either subsample. However, the interaction between social media use and CLI status was significant, and it appears that this interaction was mostly driven by a negative (albeit nonsignificant) association in the CLI subsample. Fourth, use of community sources was positively associated with misinformation endorsement in both subsamples, suggesting potential risk of gaining COVID-19 information from these sources for both CLI and non-CLI individuals.

Among the sociodemographic and political background variables, we saw a similar pattern between the CLI and non-CLI groups. Younger age, lower education, being non-Hispanic Black (vs. White), and leaning Republican in political orientation were associated with greater misinformation endorsement for both groups. The only difference is that Hispanic/Latinx/e individuals were more likely than White individuals to endorse misinformation among non-CLI individuals, whereas among CLI individuals there was no difference between the two racial/ethnic groups.

To the best of the authors’ knowledge, this study is the first to examine the prevalence of COVID-19 misinformation and its relationship with information source usage among the CLI population, identified through a nationally representative sample. Our results indicate that CLI communities are indeed more vulnerable to COVID-19 misinformation compared to non-CLI

segments of the general population. Although not all misinformation is equally detrimental [67], the nature of the misinformation assessed in the current study suggests a real possibility of deleterious consequences. For example, rushed vaccine development was the most widely endorsed misinformation in this study. This is a common myth about the COVID-19 vaccines [43] and has been linked to vaccine hesitancy in both observational and experimental studies [2,3]. Clearly, misinformation such as this can have a disproportional impact on CLI communities, adding to their already heavy burden of health disparities during the pandemic. How to protect the CLI population from the influence of detrimental misinformation is a question of significance and urgency.

Our data on information sources provide useful insights into both the dissemination pathways of misinformation and potential channels to use for remediation. First, we note that government and scientific sources are not only underutilized by CLI individuals, but also unhelpful in the mitigation of misinformation within this population. We suspect that this dual deficit has much to do with the deep-rooted distrust in government and medical establishment among CLI communities. Indeed, one of the most pronounced themes in the existing literature on CLI-related health disparities [47,59,63,64], as well as in our own qualitative research [65], is the lack of trust in information disseminated by government agencies and public health institutions. Thus, even though government and public health messaging is perhaps less likely to be influenced by misinformation, it often fails to reach and engage the CLI population. As a result, government and scientific sources may be marginalized in the fight against misinformation in CLI communities.

Mainstream and news media appear to have played a protective role against misinformation in the CLI population. This finding is consistent with some previous research

that showed exposure to traditional media to be associated with lower beliefs in conspiracy theories and misinformation [54]. Broadcast and cable TV, in particular, are widely used by CLI individuals for COVID-19 information. Although the content featured in these media outlets is necessarily complex and likely influenced by the political leaning of specific channels, the fact that they have significant reach and a negative association with misinformation endorsement among CLI communities suggests that they may be a productive venue for both the dissemination of accurate information and strategic messaging aimed at misinformation correction. Social media, on the other hand, have no clear relationship with misinformation for either the CLI or non-CLI population, although the trend in the CLI data points to the possibility that social media may have been a mitigating force when it comes to misinformation in this population. These findings are interesting because social media are often blamed for the spread of misinformation during the pandemic [66,68,69]. Our data suggest that broad conclusions may be premature without diving more deeply into the dynamic, countervailing influences of factual versus false information on these vast platforms [70].

A positive association between the use of community sources and misinformation endorsement appear to exist in both CLI and non-CLI communities. Although these sources are used by a relatively low number of community members, they include important social institutions such as community centers and churches. Our findings urge attention to the nature of the COVID-19 information that flows through these social hubs and greater efforts to monitor and mitigate misinformation in these community settings. Strategic partnerships with community leaders and institutions could be useful to these efforts.

This study has important implications for protecting and enhancing the well-being of the CLI population during the pandemic. First, misinformation is an important risk factor that may

exacerbate the health disparities faced by this population. Targeted efforts are needed to monitor and combat the spread and influence of misinformation in CLI communities. Second, mainstream media, particularly television, are important channels to reach and engage the CLI population. While comprehensive public health messaging may seek to leverage all available media, purposeful placement of messages in traditional media may prove especially beneficial for members of CLI communities. Third, efforts are needed to promote the use of government and scientific sources among the CLI population. A focal point in these efforts will be to build trust through messages that acknowledge past and ongoing mistreatments, validate feelings of neglect, mistrust, and betrayal, and express willingness to work hard to regain the CLI communities' confidence in government agencies and public health institutions. Fourth, and related to the point above, public health communication should identify sources that already enjoy relatively high levels of trust among CLI communities and partner with these sources as a conduit for the dissemination of accurate COVID-19 information. Finally, care needs to be taken when engaging community sources. Simply pushing accurate information into these environments may cause confusion and uncertainty as communities work to make sense of new information. Close monitoring and effective mitigation of misinformation in community settings is an important task in its own right. It is also essential for the success of broader pandemic-related health promotion efforts.

This study has several limitations. First, the list of misinformation, while carefully extracted from previous qualitative research, is necessarily time-bound, thus merely a snapshot of reality in a quickly evolving pandemic. Second, the information sources surveyed in this study did not capture the political leaning of specific outlets within the same media (e.g., Fox news versus MSNBC). This has limited the scope of our investigation. Third, source usage was

measured as a simple dichotomy in this study. The amount of usage of each source was thus unclear. Fourth, the cross-sectional nature of the current data does not support causal interpretation of the relationship between source usage and misinformation endorsement. Fifth, the study measured criminal legal system involvement as a dichotomous variable. Important differences might exist among the CLI individuals depending on the amount and type of involvement with the legal system and time since involvement. Sixth, as seen in other studies reliant on household surveys, we had a modest response rate. Nevertheless, the AmeriSpeak panel's response rate of 37% is one of the highest for comparable national probability-based household panels [71]. We weighted the data to national census benchmarks, taking into account selection probabilities and addressed possible non-response bias with statistical weights and non-response adjustments. Finally, despite a fairly large national sample, the number of CLI respondents in this study was relatively small, resulting in limited power in some of our analyses. Even with these limitations, this study contributes useful evidence to identify misinformation as an important concern for the well-being of CLI individuals during the pandemic. Efforts to contain and mitigate misinformation in this vulnerable population using appropriate information sources are warranted.

References

1. DHHS. Confronting Health Misinformation: The U.S. Surgeon General's Advisory on Building a Healthy Information Environment. U.S. Department of Health and Human Services; 2021 p. 22. Available: https://www.hhs.gov/sites/default/files/surgeon-general-misinformation-advisory.pdf

2. Loomba S, de Figueiredo A, Piatek SJ, de Graaf K, Larson HJ. Measuring the impact of COVID-19 vaccine misinformation on vaccination intent in the UK and USA. Nat Hum Behav. 2021;5: 337–348. doi:10.1038/s41562-021-01056-1

3. Ognyanova K, Lazer D, Baum MA, Druckman J, Perlis RH, Santillana M, et al. Covid-19 Vaccine Misinformation Trends. The Covid States Project; 2022 p. 24. Report No.: 82.

4. Janmohamed K, Walter N, Nyhan K, Khoshnood K, Tucker JD, Sangngam N, et al. Interventions to Mitigate COVID-19 Misinformation: A Systematic Review and Meta-Analysis. Journal of Health Communication. 2021;26: 846–857. doi:10.1080/10810730.2021.2021460

5. van der Linden S. Misinformation: susceptibility, spread, and interventions to immunize the public. Nat Med. 2022;28: 460–467. doi:10.1038/s41591-022-01713-6

6. ODPHP. Social Determinants of Health - Healthy People 2030. [cited 29 Apr 2022]. Available: https://health.gov/healthypeople/priority-areas/social-determinants-health

7. Dong KR, Must A, Tang AM, Beckwith CG, Stopka TJ. Competing priorities that rival health in adults on probation in Rhode Island: substance use recovery, employment, housing, and food intake. BMC Public Health. 2018;18: 289. doi:10.1186/s12889-018-5201-7

8. Binswanger IA, Krueger PM, Steiner JF. Prevalence of chronic medical conditions among jail and prison inmates in the USA compared with the general population. Journal of Epidemiology & Community Health. 2009;63: 912–919. doi:10.1136/jech.2009.090662

9. Bui J, Wendt M, Bakos A. Understanding and Addressing Health Disparities and Health Needs of Justice-Involved Populations. Public Health Rep. 2019;134: 3S-7S. doi:10.1177/0033354918813089

10. NIDA. Criminal Justice DrugFacts. 1 Jun 2020 [cited 18 May 2022]. Available: https://nida.nih.gov/publications/drugfacts/criminal-justice

11. Winkelman TNA, Chang VW, Binswanger IA. Health, Polysubstance Use, and Criminal Justice Involvement Among Adults With Varying Levels of Opioid Use. JAMA Network Open. 2018;1: e180558. doi:10.1001/jamanetworkopen.2018.0558

12. Bronson J, Berzofsky M. Indicators of Mental Health Problems Reported by Prisoners and Jail Inmates, 2011-12. Bureau of Justice Statistics; 2017. Report No.: NCJ 250612.

13. Feucht TE, Gfroerer J. Mental and Substance Use Disorders among Adult Men on Probation or Parole: Some Success against a Persistent Challenge. Office of Justice Programs; p. 17. Report No.: NCJ235637.

14. Steadman HJ, Peters RH, Carpenter C, Mueser KT, Jaeger ND, Gordon RB, et al. Six Steps to Improve Your Drug Court Outcomes for Adults with Co-Occurring Disorders. Drug Court Practitioner Fact Sheet. 2013;VIII: 1–28.

15. Steadman HJ, Osher FC, Robbins PC, Case B, Samuels S. Prevalence of Serious Mental Illness Among Jail Inmates. PS. 2009;60: 761–765. doi:10.1176/ps.2009.60.6.761

16. Vaughn MG, DeLisi M, Beaver KM, Perron BE, Abdon A. Toward a criminal justice epidemiology: Behavioral and physical health of probationers and parolees in the United States. Journal of Criminal Justice. 2012;40: 165–173. doi:10.1016/j.jcrimjus.2012.03.001

17. Montoya-Barthelemy AG, Lee CD, Cundiff DR, Smith EB. COVID-19 and the Correctional Environment: The American Prison as a Focal Point for Public Health. American Journal of Preventive Medicine. 2020;58: 888–891. doi:10.1016/j.amepre.2020.04.001

18. Abraham LA, Brown TC, Thomas SA. How COVID-19's Disruption of the U.S. Correctional System Provides an Opportunity for Decarceration. Am J Crim Just. 2020;45: 780–792. doi:10.1007/s12103-020-09537-1

19. Alohan D, Calvo M. COVID-19 Outbreaks at Correctional Facilities Demand a Health Equity Approach to Criminal Justice Reform. J Urban Health. 2020;97: 342–347. doi:10.1007/s11524-020-00459-1

20. Mukherjee TI, El-Bassel N. The perfect storm: COVID-19, mass incarceration and the opioid epidemic. International Journal of Drug Policy. 2020;83: 102819. doi:10.1016/j.drugpo.2020.102819

21. UCLA Law COVID Behind Bars Data Project. [cited 29 Apr 2022]. Available: https://uclacovidbehindbars.org/

22. Novisky MA, Nowotny KM, Jackson DB, Testa A, Vaughn MG. Incarceration as a Fundamental Social Cause of Health Inequalities: Jails, Prisons and Vulnerability to COVID-19. The British Journal of Criminology. 2021;61: 1630–1646. doi:10.1093/bjc/azab023

23. Okonkwo NE, Aguwa UT, Jang M, Barré IA, Page KR, Sullivan PS, et al. COVID-19 and the US response: accelerating health inequities. BMJ Evidence-Based Medicine. 2021;26: 176–179. doi:10.1136/bmjebm-2020-111426

24. Bowleg L. We're Not All in This Together: On COVID-19, Intersectionality, and Structural Inequality. Am J Public Health. 2020;110: 917–917. doi:10.2105/AJPH.2020.305766

25. Williams DR, Cooper LA. Reducing Racial Inequities in Health: Using What We Already Know to Take Action. International Journal of Environmental Research and Public Health. 2019;16: 606. doi:10.3390/ijerph16040606

26. Williams DR, Cooper LA. COVID-19 and Health Equity—A New Kind of "Herd Immunity." JAMA. 2020;323: 2478–2480. doi:10.1001/jama.2020.8051

27. Aliyu S, Travers JL, Norful AA, Clarke M, Schroeder K. The Lived Experience of Being Diagnosed With COVID-19 Among Black Patients: A Qualitative Study. Journal of Patient Experience. 2021;8: 2374373521996963. doi:10.1177/2374373521996963

28. Cooper LA, Williams DR. Excess Deaths From COVID-19, Community Bereavement, and Restorative Justice for Communities of Color. JAMA. 2020;324: 1491–1492. doi:10.1001/jama.2020.19567

29. Galea S, Abdalla SM. COVID-19 Pandemic, Unemployment, and Civil Unrest: Underlying Deep Racial and Socioeconomic Divides. JAMA. 2020;324: 227–228. doi:10.1001/jama.2020.11132

30. Hill L, Artiga S. COVID-19 Cases and Deaths by Race/Ethnicity: Current Data and Changes Over Time. In: KFF [Internet]. 22 Feb 2022 [cited 29 Apr 2022]. Available: https://www.kff.org/coronavirus-covid-19/issue-brief/covid-19-cases-and-deaths-by-race-ethnicity-current-data-and-changes-over-time/

31. Schneider JA, Taylor BG, Hotton AL, Lamuda PA, Ozik J, Lin Q, et al. National variability in Americans' COVID-19 protective behaviors: Implications for vaccine roll-out. PLOS ONE. 2021;16: e0259257. doi:10.1371/journal.pone.0259257

32. World Health Organization. Managing the COVID-19 infodemic: Promoting healthy behaviours and mitigating the harm from misinformation and disinformation. 2020 [cited 3 Apr 2022]. Available: https://www.who.int/news/item/23-09-2020-managing-the-covid-19-infodemic-promoting-healthy-behaviours-and-mitigating-the-harm-from-misinformation-and-disinformation

33. Krause NM, Freiling I, Beets B, Brossard D. Fact-checking as risk communication: the multi-layered risk of misinformation in times of COVID-19. Journal of Risk Research. 2020;0: 1–8. doi:10.1080/13669877.2020.1756385

34. Vosoughi S, Roy D, Aral S. The spread of true and false news online. Science. 2018;359: 1146–1151. doi:10.1126/science.aap9559

35. Mian A, Khan S. Coronavirus: the spread of misinformation. BMC Medicine. 2020;18: 89. doi:10.1186/s12916-020-01556-3

36. Islam MS, Kamal A-HM, Kabir A, Southern DL, Khan SH, Hasan SMM, et al. COVID-19 vaccine rumors and conspiracy theories: The need for cognitive inoculation against misinformation to improve vaccine adherence. PLOS ONE. 2021;16: e0251605. doi:10.1371/journal.pone.0251605

37. Greene CM, Murphy G. Quantifying the effects of fake news on behavior: Evidence from a study of COVID-19 misinformation. Journal of Experimental Psychology: Applied. 2021;27: 773–784. doi:10.1037/xap0000371

38. Lee JJ, Kang K-A, Wang MP, Zhao SZ, Wong JYH, O'Connor S, et al. Associations Between COVID-19 Misinformation Exposure and Belief With COVID-19 Knowledge and Preventive Behaviors: Cross-Sectional Online Study. Journal of Medical Internet Research. 2020;22: e22205. doi:10.2196/22205

39. Pickles K, Cvejic E, Nickel B, Copp T, Bonner C, Leask J, et al. COVID-19 Misinformation Trends in Australia: Prospective Longitudinal National Survey. Journal of Medical Internet Research. 2021;23: e23805. doi:10.2196/23805

40. Luk TT, Zhao S, Weng X, Wong JY-H, Wu YS, Ho SY, et al. Exposure to health misinformation about COVID-19 and increased tobacco and alcohol use: a population-based survey in Hong Kong. Tobacco Control. 2020 [cited 15 Sep 2020]. doi:10.1136/tobaccocontrol-2020-055960

41. Dhawan D, Bekalu M, Pinnamaneni R, McCloud R, Viswanath K. COVID-19 News and Misinformation: Do They Matter for Public Health Prevention? Journal of Health Communication. 2021;26: 799–808. doi:10.1080/10810730.2021.2010841

42. Lin S. Generalized anxiety disorder during COVID-19 in Canada: Gender-specific association of COVID-19 misinformation exposure, precarious employment, and health behavior change. Journal of Affective Disorders. 2022;302: 280–292. doi:10.1016/j.jad.2022.01.100

43. Hotez P, Batista C, Ergonul O, Figueroa JP, Gilbert S, Gursel M, et al. Correcting COVID-19 vaccine misinformation: Lancet Commission on COVID-19 Vaccines and Therapeutics Task Force Members*. EClinicalMedicine. 2021;33. doi:10.1016/j.eclinm.2021.100780

44. Chung M, Jones-Jang SM. Red Media, Blue Media, Trump Briefings, and COVID-19: Examining How Information Sources Predict Risk Preventive Behaviors via Threat and Efficacy. Health Communication. 2021;0: 1–8. doi:10.1080/10410236.2021.1914386

45. Jamieson KH, Albarracín D. The relation between media consumption and misinformation at the outset of the SARS-CoV-2 pandemic in the US. Harvard Kennedy School Misinformation Review. 2020;1. doi:10.37016/mr-2020-012

46. Khorasani SB, Koutoujian PJ, Zubiago J, Guardado R, Siddiqi K, Wurcel AG. COVID-19 Vaccine Interest among Corrections Officers and People Who Are Incarcerated at Middlesex County Jail, Massachusetts. J Urban Health. 2021;98: 459–463. doi:10.1007/s11524-021-00545-y

47. Lessard D, Ortiz-Paredes D, Park H, Varsaneux O, Worthington J, Basta NE, et al. Barriers and facilitators to COVID-19 vaccine acceptability among people incarcerated in Canadian federal prisons: A qualitative study. Vaccine: X. 2022;10: 100150. doi:10.1016/j.jvacx.2022.100150

48. Viswanath K, Lee EWJ, Pinnamaneni R. We Need the Lens of Equity in COVID-19 Communication. Health Communication. 2020;35: 1743–1746. doi:10.1080/10410236.2020.1837445

49. Brodie M, Hamel EC, Altman DE, Blendon RJ, Benson JM. Health news and the American public, 1996–2002. Journal of Health Politics, Policy and Law. 2003;28: 927–950. doi:10.1215/03616878-28-5-927

50. Brown JD, Witherspoon EM. The mass media and American adolescents' health. Journal of Adolescent Health. 2002;31: 153–170.

51. Frost RL, Rickwood DJ. A systematic review of the mental health outcomes associated with Facebook use. Computers in Human Behavior. 2017;76: 576–600. doi:10.1016/j.chb.2017.08.001

52. Brennen JS, Simon FM, Howard PN, Nielsen RK. Types, Sources, and Claims of COVID-19 Misinformation. Reuters Institute for the Study of Journalism; 2020 p. 13.

53. Li S, Feng B, Liao W, Pan W. Internet Use, Risk Awareness, and Demographic Characteristics Associated With Engagement in Preventive Behaviors and Testing: Cross-Sectional Survey on COVID-19 in the United States. Journal of Medical Internet Research. 2020;22: e19782. doi:10.2196/19782

54. De Coninck D, Frissen T, Matthijs K, d'Haenens L, Lits G, Champagne-Poirier O, et al. Beliefs in conspiracy theories and misinformation about COVID-19: Comparative perspectives on the role of anxiety, depression and exposure to and trust in information sources. Frontiers in Psychology. 2021;12. doi:10.3389/fpsyg.2021.646394

55. Gerosa T, Gui M, Hargittai E, Minh Hao Nguyen. (Mis)informed During COVID-19: How Education Level and Information Sources Contribute to Knowledge Gaps. International Journal of Communication (19328036). 2021;15: 2196–2217.

56. Kim Y. Trust in health information websites: A systematic literature review on the antecedents of trust. Health Informatics J. 2016;22: 355–369. doi:10.1177/1460458214559432

57. Sbaffi L, Rowley J. Trust and Credibility in Web-Based Health Information: A Review and Agenda for Future Research. J Med Internet Res. 2017;19: e218. doi:10.2196/jmir.7579

58. Armstrong K, McMurphy S, Dean LT, Micco E, Putt M, Halbert CH, et al. Differences in the Patterns of Health Care System Distrust Between Blacks and Whites. J GEN INTERN MED. 2008;23: 827–833. doi:10.1007/s11606-008-0561-9

59. Boulware LE, Cooper LA, Ratner LE, LaVeist TA, Powe NR. Race and trust in the health care system. Public health reports. 2003;118: 358–365.

60. Gilson L. Trust and the development of health care as a social institution. Social Science and Medicine. 2003;56: 1453–1468.

61. Portillo S, Goldberg V, Taxman FS. Mental Health Peer Navigators: Working With Criminal Justice–Involved Populations. The Prison Journal. 2017;97: 318–341. doi:10.1177/0032885517704001

62. Schuster B, Britton H, Seo H, Altschwager D, Booton E, Ault M, et al. "I Don't Just Take Whatever They Hand to Me": How Women Recently Released from Incarceration Access Internet Health Information. Women & Criminal Justice. 2022;0: 1–17. doi:10.1080/08974454.2022.2040692

63. Lorvick J, Hemberg J, Cropsey K, Wickliffe J, Faust A, Comfort M, et al. Sources of Information and Health Care Experiences Related to COVID-19 among Women Involved in Criminal Legal System in Three U.S. Cities. Arch Women Health Care. 2020;3. doi:10.31038/awhc.2020351

64. Krishnan L, Ogunwole SM, Cooper LA. Historical Insights on Coronavirus Disease 2019 (COVID-19), the 1918 Influenza Pandemic, and Racial Disparities: Illuminating a Path Forward. Ann Intern Med. 2020;173: 474–481. doi:10.7326/M20-2223

65. Davidson-Mhonde RR, Riddick BR, Shaw CC, Hingle A, Rudes DS, Schneider J, et al. “I just don’t know what to believe”: Sensemaking during the COVID-19 Pandemic among Criminal Legal Involved Communities. under review.

66. Jemielniak D, Krempovych Y. An analysis of AstraZeneca COVID-19 vaccine misinformation and fear mongering on Twitter. Public Health. 2021;200: 4–6. doi:10.1016/j.puhe.2021.08.019

67. Southwell BG, Niederdeppe J, Cappella JN, Gaysynsky A, Kelley DE, Oh A, et al. Misinformation as a Misunderstood Challenge to Public Health. American Journal of Preventive Medicine. 2019;57: 282–285. doi:10.1016/j.amepre.2019.03.009

68. Jennings W, Stoker G, Bunting H, Valgarðsson VO, Gaskell J, Devine D, et al. Lack of Trust, Conspiracy Beliefs, and Social Media Use Predict COVID-19 Vaccine Hesitancy. Vaccines. 2021;9: 593. doi:10.3390/vaccines9060593

69. Lee J, Choi J, Britt RK. Social media as risk-attenuation and misinformation-amplification station: How social media interaction affects misperceptions about covid-19. Health Communication. 2021 [cited 4 Apr 2022]. doi:10.1080/10410236.2021.1996920

70. Rosenberg H, Syed S, Rezaie S. The Twitter pandemic: The critical role of Twitter in the dissemination of medical information and misinformation during the COVID-19 pandemic. Canadian Journal of Emergency Medicine. 2020; 1–4. doi:10.1017/cem.2020.361

71. Montgomery R, Dennis JM, Ganesh N. Response rate calculation methodology for recruitment of a two-phase probability-based panel: the case of AmeriSpeak. Chicago, IL: NORC at the University of Chicago; 2016.
