# Supplementary material for: Endorsement of COVID-19 misinformation among criminal legal involved individuals in the United States: Prevalence and relationship with information sources
Source: PLoS One. 2024 Jan 5;19(1):e0296752. doi: 10.1371/journal.pone.0296752 (PMC10769018; doi:10.1371/journal.pone.0296752)
Supplement: Attachment — Submitted filename: Final Response to Reviewers.docx [file pone.0296752.s003.docx]

**Response to Reviewers**

We thank the reviewers for their feedback and suggestions. Engaging with their insights has been a pleasure and made our manuscript stronger. In what follows, we respond to the reviewers’ comments point by point and describe, where appropriate, how their concerns have been addressed in the revised manuscript. Reviewers’ original comments are copied below, and our responses are marked by **AR:** (author response) and italicized. Page numbers referenced in responses are based on the manuscript file with track changes.

Reviewer #1:

This paper investigates the differences between a sample of Americans and a subsample of individuals with prior history of criminal legal system involvement (CLI) regarding their endorsement of COVID-19 misinformation and its relation to media use patterns. Using survey questionnaires and Poisson regression analyses, the authors find a number of differences, and also some similarities, between the two groups. Most notably, CLI persons show a greater frequency of misinformation endorsement than the general populace. Furthermore, some media use types were related to endorsement.

This is an interesting study, the paper is clearly written, and the interpretation of the results is generally warranted. I can see this study offering a valuable contribution to the literature on misinformation endorsement, especially by providing a more detailed look at how membership of specific social subgroups may affect how media are used and how this is connected to developing misperceptions. Before publication, however, I have a few questions and suggestions, which I will list below. Some related to how the paper is written, others are related to the analyses and their interpretations.

***AR:*** *Thank you for your positive assessment of our work. We appreciate your questions and suggestions.*

Disclaimer: Although I am a social scientist, and the methodology presented here is largely familiar to me, I cannot claim to be well-versed in the specific analyses strategies used here, especially the Poisson regression.

COMMENTS:
1. One of my chief concerns is to do with the data, more specifically the extremely different sample sizes of the main and subsample, with the general sample (N=1161) forming around 9-10 times the size of the subsample (N=168). This would increase the chances of (very) unequal variances, and associated problems with over or underestimating effect p-values. I wonder what you think of this matter, and would suggest that you address it in the paper, by offering a bit more elaborate explanation of the decisions involved in this regard, as well as informing the reader about the variances in the two samples.

***AR:*** *The reviewer’s point is well taken. Unequal sample sizes are a common challenge when working with marginalized populations. Since AmeriSpeak is a nationally representative survey with no oversampling of targeted groups, it is inevitable that the subsample for CLI individuals will be much smaller than that of their non-CLI counterparts. In our study, the CLI sample size was 168, about 15% of the total N. This percentage is comparable to that of important race/ethnicity minority groups in nationally representative samples that are often subjected to comparisons against larger groups. We have carefully considered sample size restrictions in model building (more on this in response to comment #2 below) and added a brief discussion on these considerations in the analysis section (p.15).*

*We also acknowledge that, as the reviewer pointed out, unequal sample sizes have implications for power and precision of estimation. We have noted this as a limitation in the discussion and call for future research to oversample CLI individuals to enable more balanced analysis and address this concern raised by Reviewer 1 (p.32).*

*We provided the standard deviation of number of misinformation statements endorsed by the CLI vs. non-CLI groups in Table 2. They were not very different in size (1.34 vs. 1.87).*

2. Relatedly, as you rightly acknowledge in the discussion section (p. 29), because of the small CLI sample, there are some issue with the power of the analyses. Could you expand a little more on that in the paper? I.e., while you acknowledge the problem, you do not really offer a comforting rebuttal. Moreover, there is no information about a priori or post hoc power analyses performed. One thing is that one could argue that the analyses should be powered on the smallest sample, i.e. N=168. Would this have consequences for your results and their interpretation, also with regards to the fairly modest effect sizes (see below)?

***AR:*** *We thank the reviewer for encouraging us to be thoughtful about the power issue in our data. We have been careful not to build overly complex models in our analyses considering the relatively modest CLI sample size. Our CLI-specific model (Table 5), for example, included 16 predictors, in line with the widely used conventional guideline of 10 cases per variable in regression analysis (although methodological research has shown that much lower case to variable ratio could still produce adequate estimation of regression coefficients, standard errors, and confidence intervals; Austin & Steyerberg, 2015; Vittinghoff & McCulloch, 2007). We have added a point about managing model complexity in the analysis strategy section (p.15).*

*We did not perform a priori power analysis because there is no prior research we can draw on to inform potential effect sizes. Given that this study was fielded as part of a large national survey, there was little we could do to improve the power of our analysis. On the other hand, even with a modest sample, we uncovered some important differences between CLI and non-CLI groups, which suggests that continued attention to misinformation among CLI communities is warranted. We do hope that future research, either by us or by other research teams, can find ways to generate larger CLI samples to enable more powerful and more balanced analysis.*

*Regarding effect sizes, please see our response to a related comment below (#4).*

3. Regarding the interpretations of the results, I noticed that there are many instances of the “there was a difference/correlation between x and y, but it was not significant” variant. I would suggest that it would be more correct to state that when you did not find a significant difference/correlation, there is no difference/correlation, without qualification.

***AR:*** *Thank you for pointing this out. We have removed all such references in the manuscript.*

4. In my opinion, the fact that most effect sizes are in the small-moderate range, deserves more attention. I.e., there are some differences between the two groups, but the differences are perhaps not that dramatic. Also considering the fact that most misinformation statements exhibit fairly low endorsement.

***AR:*** *This is an important observation. We have added a paragraph (p.26) to acknowledge the low endorsement levels and relatively modest differences between CLI and non-CLI groups on some of the misinformation items. We also noted that the levels of endorsement observed in our study were comparable to those reported in other national studies.*

5. Can you give us a little more information on how the subsample is or isn’t representative of the larger CLI population?

***AR:*** *The CLI population consists of 20 percent of the American adult population. Unfortunately, there are no national statistics describing this population. The existing surveys such as the National Survey on Drug Use and Health sponsored by SAMSHA only asks a question about last year arrest or whether the person is currently on probation and/or parole. This question does not cover the full range of the CLI population since it does not include individuals that are detained in jail and/or prison or a person that has had a history of involvement in the criminal legal system. There are surveys that are conducted by the Bureau of Justice Statistics (BJS), but they are specific to various legal conditions—jail, prison, and/or probation—and these are generally dated and it is not possible to aggregate these findings across surveys because the timing of the survey (from 1995 to 2014), the type of questions, and the samples are not consistent.*

*On the surface the CLI subsample in our study approximates the percentage of the general population that are justice involved (15% compared to 20% of the general population).*

*Given that the AmeriSpeak sample was generated through probability-based sampling, we would like to think that a certain level of representativeness in subsamples can be safely assumed. But it is difficult to evaluate in precise terms how much our CLI subsample is representative of all CLI individuals in the U.S.*

6. Was there any factor analysis done on the misinfo statements? I ask partly because one of the items seems a bit of an outlier, representing more of a conspiracy thinking than purely misconceptions: “COVID-19 is a scheme for rich people and big companies to make money off of the testing and vaccines.”

***AR:*** *We didn’t conduct any factor analysis for the misinformation items because we did not treat them as indicators of a latent construct. Instead, we created a simple index to see how many of these statements were endorsed by each respondent. Misinformation has diverse origin, form, content focus, and level of sophistication. Existing research rarely takes a latent variable approach to it. We agree that the statement singled out by the reviewer has more of a conspiracy thinking flavor to it. There might be interesting differences between this item and others. We will keep this possibility in mind for future research.*

7. The introduction to the paper is very thorough (p3-8). In fact, it is a little overwhelming in its detail. I would say that the paper would be improved if the introduction would be a little more concise, and many of the arguments made would be equally strong if the data and findings from prior research could be presented in a more summarized form. I for me found it sometimes difficult to stay focused through the multitude of percentages presented.

***AR:*** *Thank you for the suggestion. We have edited the introduction to make it more concise and streamlined, especially on p.4, where we cited a lot of statistics in the original draft.*

8. Likewise, in my opinion there is no need to repeat all the findings in the discussion, because we can all read it in the results section. Just a quick summary of the most important results, and then on to the interpretation of the results.

***AR:*** *Another great suggestion. Thank you. We have edited the summary of findings in the discussion section to make it more succinct (p.25).*

9. This would also free up some room for two of my other suggestions. Firstly, I would like to hear a bit more about the scientific contribution or relevance of focusing on this particular group. As it stands, the focus is much more on the societal relevance. Secondly, I wonder if the paper and its scientific contribution would not be stronger if you tried to find a theoretical framework, from which you could derive testable hypotheses? Now, the paper leaves the reader with the question of what it all means. This could be addressed both in the introduction and discussion section, the latter of which could use some more engagement with prior research, and especially extant theoretical notions in the field.

***AR:*** *We have added a paragraph in the discussion section (p.30) to address the scientific contributions of the study. As an initial effort to examine the intersection of CLI and COVID misinformation, this study was not designed to test any specific theory. But its findings can certainly inform future theory-driven research in this area. We briefly speculated on these possibilities in the added paragraph.*

Minor things:
1. Table 1: for the reader’s convenience, could you provide info on the meaning of the p-values, either in the column or below the table in a note?

***AR:*** *We added a note on where the P-values came from.*

2. In Table 5 it says ‘oder age’, which should be ‘older’ I guess. But reading the text, I got confused, because there It says that younger age associated with misinformation endorsement (p23). This does not seem to correspond with what’s in the table for older age. Or did I misread?

***AR:*** *Thanks for catching this error. It should read “older” in both the table and the text. We have made the corrections (pp.24-25).*

Thanks for allowing me to read this paper, and the best of luck with it!

***AR:*** *Thank you very much!*

Reviewer #2:

The article investigates the prevalence of COVID-19 related misinformation and its relationship with COVID-19 information sources used among Americans experiencing CLI. The study surveyed a nationally representative sample of American adults aged 18+ including a subsample of CLI individuals. The results show that CLI participants endorsed a greater number of misinformation statements and reported less use of government and scientific sources and personal sources for COVID-19 information compared to non-CLI participants. The study suggests that building trust in important information sources is critical to the containment and mitigation of COVID-related misinformation in the CLI population.

Although the authors claimed that this study is the first to focus on individuals experiencing CLI, the findings fail to offer any significant new insights into the existing knowledge in the field. Despite using a nationally representative sample, the sample size for the CLI population may be considered too small to generate reliable and robust results. Additionally, the use of Poisson regression models in the analysis may oversimplify the complex ways in which misinformation can impact individuals and fails to fully consider the contextual factors that contribute to its spread.

***AR:*** *We acknowledge that the subsample of CLI respondents in this study was modest in size. As noted in our earlier response to Reviewer 1, this is a common challenge when using nationally representative data to study a marginalized population. Unless there is effort to oversample the target population (which was not an option for this study as the larger survey was not designed for the sole purpose of our research), there will always be imbalance in sample size between the marginalized population and the rest of the general population. Despite a relatively modest CLI sample, our study revealed systematic and meaningful differences between the CLI and non-CLI individuals. Where significant effects were detected, the confidence intervals were all relatively narrow, suggesting reasonable precision of the estimates.*

*Although we agree that the current findings should be considered with care in light of the modest CLI sample size, we do think that our work has contributed important new insights to the literature. Greater endorsement of COVID misinformation among the CLI individuals reveals a new dimension of health and informational disparity that disenfranchises this population. The associations between misinformation endorsement and usage of information sources point to important mechanisms for both the spread and potential mitigation of misinformation. These insights, as discussed in the manuscript, have important implications for both research and practice.*

*We used Poisson regression for the main analysis in this study because we were interested primarily in the breadth of misinformation endorsement in the CLI (and non-CLI) communities. The reviewer is correct that misinformation can arise and impact individuals in complex ways. Each misconception has its own intricacies. Research can delve deeply into the origin, spread, and impact of specific misinformation beliefs. But there is also value in monitoring and understanding the number and range of misinformation beliefs within a given population. Our work is primarily an effort on the latter front. Other influential work on misinformation during the pandemic has taken a similar approach* (e.g., Jamieson & Albarracín, 2020; Ognyanova et al., 2022)*.*

*As a final note, we wish to point out that we used logistic regression to examine the role of CLI in endorsing specific misinformation items (Table 2). We observed consistent differences between CLI and non-CLI groups on the individual items that were also consistent with what we saw in the Poisson regression model. To conserve space, we did not present full details of the models but the coefficients for CLI were summarized in Table 2.*

The authors need to revise the literature review especially in the "CLI as social determinants of health" subsection to ensure that it is more relevant and directly tied to the goals and objectives of the study. The literature review should clearly and effectively support the authors' argument and provide a comprehensive understanding of the current knowledge and research on CLI as a social determinant of health. Additionally, the literature review should highlight the gap in the existing knowledge that the authors aim to address through their study.

***AR:*** *We have revised the section on CLI as a social determinant of health (pp.3-5), based on guidance from both Reviewers 1 and 2, to strengthen the connection between this idea and the objectives of the current study. These changes also highlight the lack of attention to issues related to health information and misinformation in the CLI population as an important gap in the existing literature.*

Although the authors mentioned that the data is available, they did not provide the link to the platform where the data underlying the findings in the manuscript can be accessed.

***AR:*** *The data is currently available upon request by contacting Dr. Bruce Taylor via email at* [*taylor-bruce@norc.org*](mailto:taylor-bruce@norc.org)*.*

Reviewer #3: The paper was written in clear and simple language, which will benefit the non-scientific/academic reader. While a few sentences and clauses were unclear and perhaps could be revised to provide a much clearer explanation, I found the paper acceptable. I am not an expert in the quantitative methodology, therefore will not be able to comment on the method and analysis of the study.

***AR:*** *Thank you for your positive feedback!*

Additional comments:

Note in editor’s decision letter: In addition, substantive ideas for policies to combat misinformation was mentioned as a need in private comments by one of the reviewers.

***AR:*** *We have added a brief discussion on potential policy actions to combat misinformation (pp.31-32).*

The editor shared a PDF of the manuscript with some inserted comments. Most were about minor wording changes. We have carefully considered the suggestions and revised the manuscript accordingly.

P.3, L.50. “To the best of our knowledge,…” Opinion based. remove this unless the author has exhaustively conduct meta-analysis to support this.

***AR:*** *The phrase has been removed.*

P.3, L.54-56. This sentence came off as an afterthought. Revise to dictate its importance despite secondary in nature.

***AR:*** *We have revised the sentence to justify the importance of this aim of the study.*

P.3, L.62-64. Confusing sentence. Revise.

***AR:*** *We have revised the sentence to improve its clarity.*

P.4, L.79-82. Old study. Revise and replace to a newer study.

***AR:*** *We have replaced this study with a newer one that has used 13 years of data from the National Survey on Drug Use and Health to demonstrate the same trends.*

P.6, L.118. Citation

***AR:*** *Thanks for catching this error. We have converted the citation to the Plos One style.*

P.8, L.167-168. Problem statement.

***AR:*** *We were unsure what the comment means. Since this sentence is not essential, we have deleted it in the revised manuscript.*

P.9, L.188. what's a liberal sources?

***AR:*** *We have revised the wording and added an example for clarity.*

There were two highlighted sentences on page 10 with no comments. We assume the reviewer had no concerns about those sentences.

We thank the reviewers again for their great comments and suggestions. They have helped to make this manuscript stronger. We hope the reviewers and editor will find our revision and response satisfactory.

**References**

Austin, P. C., & Steyerberg, E. W. (2015). The number of subjects per variable required in linear regression analyses. *Journal of Clinical Epidemiology*, *68*(6), 627–636. https://doi.org/10.1016/j.jclinepi.2014.12.014

Jamieson, K. H., & Albarracín, D. (2020). The relation between media consumption and misinformation at the outset of the SARS-CoV-2 pandemic in the US. *Harvard Kennedy School Misinformation Review*, *1*(3). https://doi.org/10.37016/mr-2020-012

Ognyanova, K., Lazer, D., Baum, M. A., Druckman, J., Perlis, R. H., Santillana, M., Qu, H., Trujillo, K. L., Safarpour, A. C., Uslu, A., Quintana, A., Green, J., Pippert, C., & Shere, A. (2022). *Covid-19 Vaccine Misinformation Trends* (No. 82; The Covid States Project: A 50-State Covid-19 Survey, p. 24). The Covid States Project.

Vittinghoff, E., & McCulloch, C. E. (2007). Relaxing the rule of ten events per variable in logistic and Cox regression. *American Journal of Epidemiology*, *165*(6), 710–718. https://doi.org/10.1093/aje/kwk052
