# Supplementary material for: Endorsement of COVID-19 misinformation among criminal legal involved individuals in the United States: Prevalence and relationship with information sources
Source: PLoS One. 2024 Jan 5;19(1):e0296752. doi: 10.1371/journal.pone.0296752 (PMC10769018; doi:10.1371/journal.pone.0296752)
Supplement: Attachment — Submitted filename: Response to Reviewers R2.docx [file pone.0296752.s004.docx]

**Response to Reviewers**

We thank the reviewers for their feedback on our revised manuscript. In what follows, we respond to the reviewers’ comments point by point and describe, where applicable, revisions undertaken following their suggestions. Reviewers’ original comments are copied below, and our responses are marked by **AR:** (author response) and italicized.

Reviewer #1: I find the authors have addressed my comments adequately. I have two remaining suggestions:
1. Re. comment 2 on the power issues: your addition to the analyses section is sufficient, however, I would recommend you add 1-2 references to back up your statement about the cases per variable ratio

**AR**: *Thank you. We have added references to justify the cases per variable ratio.*

2. Re comment 5: I recommend incorporating your response (perhaps slightly abbreviated) into the sample characteristics paragraph, or alternatively into an endnote, as I do not think this information is directly apparent to the uninitiated

**AR**: *Thank you. We have incorporated a brief version of our response into the sample characteristics paragraph.*

Reviewer #4: The research is significant due to numerous distinct factors: The research contributes to understanding misinformation as a form of disparity affecting CLI communities during the pandemic. It specifically targets criminally legal-involved (CLI) communities, a group often overlooked in misinformation research.
Additionally, the study examines both the endorsement of COVID-19 misinformation and the relationship with various information sources in CLI communities.
Finally, the paper employs detailed logistic and Poisson regression analyses, enhancing the reliability and depth of the findings with a detailed misinformation analysis.

**AR**: *Thank you for the positive feedback on our work.*

Reviewer #5: Please include the -19 in the COVID to be more specific.

**AR**: *Thank you for this reminder. We now consistently refer to COVID-19 in the manuscript.*

Please consider improving your entire topic also to make it clearer.
i.e.: Examining the Endorsement of COVID-19 Misinformation Among Individuals in the Criminal Legal System:….

**AR**: *We have modified our title, which now reads “Endorsement of COVID-19 misinformation among criminal legal involved individuals in the United States: Prevalence and relationship with information sources.” We did not refer to the population of interest as “individuals in the criminal legal system” because not all those with criminal legal involvement are currently “in the system.” We also added “in the United States” to make clear where the study was conducted.*

Specified each of the questions the research seeks to answer immediately after the background and the objective. This can be according to the group of analysis in the study.

**AR**: *We have added research questions (RQs) to the end of the Current Study section.*

We would like to thank the reviewers again for their great feedback. We hope they will find the changes made and our response here satisfactory.
